# Patients’ Experiences of Using a Self-help App for Posttraumatic Stress Disorder: Qualitative Study

**DOI:** 10.2196/26852

**Published:** 2021-08-04

**Authors:** Lisa H G Riisager, Anne Bryde Christensen, Frederik Bernt Scharff, Ida-Marie T P Arendt, Israa Ismail, Marianne Engelbrecht Lau, Stine Bjerrum Moeller

**Affiliations:** 1 Unit for Psychotherapy Research Stolpegaard Psychotherapy Centre Mental Health Services Capital Region of Denmark Gentofte Denmark; 2 Department of Psychology University of Southern Denmark Odense Denmark; 3 Research Unit for Psychotherapy & Psychopathology Mental Health Services West, Region Zealand Slagelse Denmark

**Keywords:** app, PTSD, mHealth, qualitative analysis, patient experience, posttraumatic stress disorder, thematic analysis, smartphone, intervention, mobile phone

## Abstract

**Background:**

Posttraumatic stress disorder (PTSD) is a common disorder that requires more treatment options. Mobile health (mHealth) app interventions are promising for patients with PTSD, as they can provide easily accessible support, strategies, and information. However, knowledge about mHealth interventions is sparse and primarily based on quantitative studies.

**Objective:**

The aim of this study is to qualitatively explore the experiences of patients with PTSD with regard to using an mHealth app as a stand-alone intervention before commencing psychotherapeutic treatment.

**Methods:**

We conducted semistructured interviews with 14 participants 6 weeks after they received the app. The participants were all referred to PTSD treatment and were waiting to commence psychotherapeutic treatment. During this waiting time, the participants had no contact with the health staff. Interviews were transcribed and were analyzed using thematic analysis.

**Results:**

A total of 3 themes were identified—the use of app, being a patient, and the overall evaluation of the app. The use of the app was described with the subtheme of habits, and the theme of being a patient included the subthemes of having negative experiences with the app and being a part of a research project. The use of the app encompassed how psychological factors and technical problems could interfere with the use of the app. The theme of being a patient depicted that the waiting time before starting treatment was long, and a subgroup of patients experienced feeling worse during this time, which they partly attributed to using the app. Several suggestions for change have been described in the overall evaluation of the app.

**Conclusions:**

The findings in this study revealed that emotional arousal influenced the use of the app and that it was difficult for participants to establish a habit of using the app, thus reflecting the importance of supporting habit formation when implementing an mHealth app in mental health care services. This study makes an important contribution to the field of mHealth research, as it revealed that some participants had negative experiences resulting from using the app, thus reflecting the potential harm of having an mHealth app without the support of a clinician. It is therefore recommended to use a blended care treatment or an approach in which mental health care professionals prescribe an mHealth app for relevant patients to avoid increased suicidal risk.

## Introduction

### Background

Posttraumatic stress disorder (PTSD) is a common disorder, with a prevalence of approximately 2% in European countries [1]. There are serious personal and societal consequences associated with PTSD, such as poor quality of life, high comorbidity, and increased use of health services [1,2]. Furthermore, PTSD is recognized as a risk factor for suicidal thoughts and behaviors and completed suicide [3,4]. Although the costs of PTSD are widely recognized, logistical and individual barriers, such as a lack of available mental health care services and negative beliefs about help-seeking, are common [5,6].

In Denmark, patients are referred to the public mental health services (MHS) by their general practitioner if a complex mental disorder is suspected. Patients are entitled to have a diagnostic interview within 30 days, but psychotherapeutic treatment may start substantially later depending on the available resources in the given clinic. As such, patients often experience waiting list periods exceeding 6 weeks before commencing treatment. During this time, the patient has minimal support from the MHS and, therefore, there is considerable interest in developing technological treatment alternatives.

Smartphones are owned by 79% of adults in European countries [7] and offer a platform with the potential to reach populations with otherwise limited access to health care [8]. Mobile health (mHealth) apps provide easy access to cost-effective treatment tools that can potentially increase treatment engagement and well-being [5,9]. There is great potential to effectively integrate apps into mental health care, as they can offer interventions, provide psychoeducation, promote self-awareness, and help overcome the self-stigmatization associated with receiving mental health care [10-12]. A systematic review and meta-analysis of the effects of self-management apps for patients with PTSD revealed that no significant difference was found between self-management app–based treatment groups and the control group on waiting list [8]. However, only 6 studies were included, leaving the quality of the evidence base low.

Although there is a growing body of quantitative research on mHealth apps as a supplement or stand-alone treatment [6,13-16], qualitative studies are surprisingly absent. To our knowledge, only 2 studies have used a qualitative approach to investigate patients’ experiences using an mHealth app for PTSD. A study on attitudes toward mHealth in a sample of veterans with either PTSD, depression, and/or an alcohol abuse disorder revealed a marked difference in the openness toward using mHealth apps depending on rurality, where rural veterans expressed more negative views than urban veterans [17]. Another study investigating the PTSD Coach app showed that participants found some coping strategies and self-assessment tools useful [18]. Consequently, it is still unclear how patients with PTSD perceive and experience using mHealth apps [8]. There is a need for future research to explore these experiences, as they can provide unique insights into compliance, engagement, adherence, as well as positive and adverse effects. With this exploratory qualitative study, we seek to fill this research gap.

### Objectives

To investigate the potential of using mHealth apps for patients with PTSD in MHS, an mHealth app named *PTSD Help* was developed for use as a stand-alone treatment and as a supplement to psychotherapeutic treatment. This study explores the following research question: how do patients diagnosed with PTSD experience the use of an app, PTSD Help, as a stand-alone treatment before psychotherapeutic treatment?

By exploring this aspect qualitatively, we aim to uncover both the experienced benefits and limitations of using the app, which can be used in the process of development and implementation of mHealth apps for PTSD in MHS.

## Methods

### Context

The study was conducted within the context of the study “The PTSD help app in a Danish PTSD population: A randomized controlled feasibility trial,” which investigated the feasibility of implementing the PTSD Help app in the Danish MHS [13]. The participants in the larger study were randomized to either a waiting list control group or a PTSD Help app treatment group. All participants received treatment for their PTSD diagnosis after 6 weeks [13]. The randomized controlled feasibility trial of the PTSD Help app study will be published in the near future. This study used a qualitative study design to explore the patients’ experiences using the app as a stand-alone treatment in the waiting period before the commencement of treatment in the MHS.

### Intervention: PTSD Help

**Figure 1:**
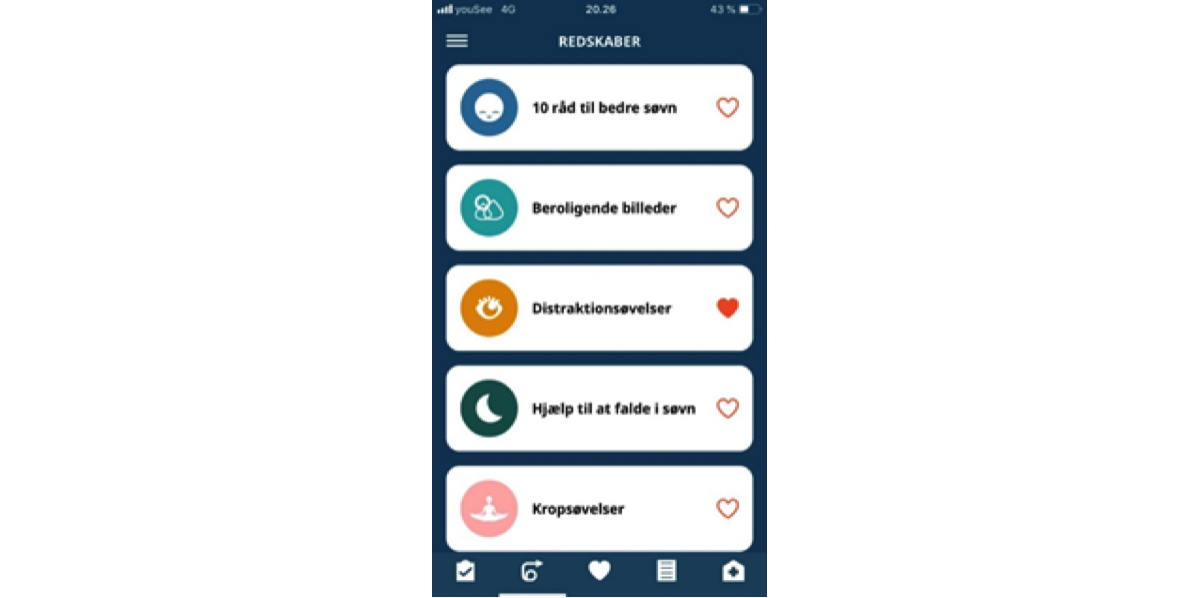
The PTSD Help app overall user surface. PTSD: posttraumatic stress disorder.

**Figure 2:**
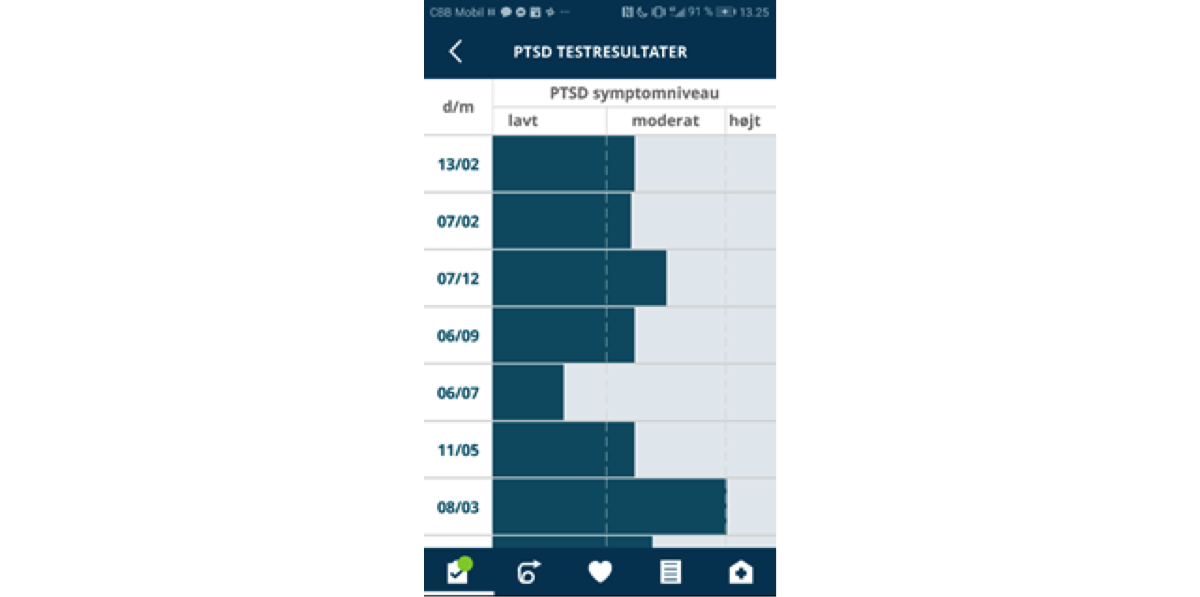
Overview of the PTSD self-assessment tool (in Danish). PTSD: posttraumatic stress disorder.

PTSD Help is an mHealth app that includes functionalities such as psychoeducation, emotion regulation tools, a note function, and a crisis plan (Figure 1). Emotion regulation tools offer a range of different interventions, including distraction exercises, grounding exercises, simple body exercises, and calming images accompanied by music. As sleep problems are common in the PTSD population, the app contains two functions to alleviate sleep problems: guided sleep meditation, in which the individual can choose between a male or female voice, and general sleep hygiene advice. Two self-assessment tools are provided in the app: the PTSD checklist for DSM-5 [19] to monitor PTSD symptoms, which is available with an interval of at least 2 weeks to be as close to the clinical use as possible. The second assessment tool aimed to monitor sleep quality (sleep condition indicator) [20], which is available daily (Figure 2). An overall favorite function is provided, giving the patient the opportunity to choose which functions are the most meaningful and bringing these functions to the front page.

To ensure that PTSD Help can be used across treatment modalities, it is designed as a generic intervention and does not include theory-specific elements. This design was chosen as the Danish MHS, which provides a range of different treatment types for patients with PTSD.

### Ethical Considerations

Participants provided both verbal and written informed consent before participating in the study and were informed that they could withdraw their consent at any time. Participants were informed that the interviews were being recorded and subsequently anonymized and transcribed verbatim. To ensure anonymity, participants were given a project ID, and audio recordings of the interviews were stored in a secure database administered by the Capital Region of Denmark. The participants’ psychotherapeutic treatment commenced shortly after the interview was conducted, thus ensuring minimal treatment gap.

### Participants

The study was conducted within the MHS of the Capital Region of Denmark (MHS-CRD), where 14 participants were recruited. Participants included in the study were aged ≥18 years, diagnosed with PTSD in accordance with the Diagnostic and Statistical Manual of Mental Disorders, Fifth Edition (DSM-5), were accepted for PTSD treatment, had access to a smartphone with iOS (version 10 or higher) or Android (version 5.0.1. or higher), and provided informed consent. The reasons for exclusion were increased suicidal risk, current episodes of bipolar disorder or psychotic disorder, current substance abuse, inability to understand and/or read Danish, and concurrent psychiatric or psychological treatment of PTSD outside the MHS-CRD [13].

### Procedure

Participants gained access to the PTSD Help app after the assessment interview and were given brief verbal general instructions on how to use the different functions in the app. This verbal introduction was based on a manuscript to ensure that all participants received the same information and took approximately 5 minutes. Furthermore, 3 days after gaining access to the app, the participants were contacted by telephone to ensure that there were no technical issues in using the app. There was no further assistance in using the app following the telephone call. Finally, the participants were contacted by telephone 6 weeks after having gained access to the app, and they were invited to participate in semistructured interviews (full description of the recruitment procedure has been provided in the study by Scharff et al [13]). The participants were not prohibited from having contact with health staff while waiting to commence psychotherapeutic treatment; however, this was rarely seen in the MHS-CRD and was therefore not monitored. Interviews were conducted in 2019. The sample was randomly selected because of its strict time schedule.

### Materials

The semistructured interview guide aimed at exploring the participants’ experiences using the PTSD Help app (see Textbox 1 for concepts covered). The interviews lasted approximately 30 minutes and were conducted over the telephone. The interviews were transcribed verbatim by a graduate psychology student.

Assessments were conducted as part of a larger study. The Mini-International Neuropsychiatric Interview 7.02. [21] was used to establish PTSD diagnosis in accordance with the DSM-5. PTSD symptoms were measured using the PTSD checklist for DSM-5 [19], which is a self-report questionnaire that assesses the presence of the four core DSM-5 PTSD symptom clusters during the past month with a cut-off score between 31 and 33, thus providing knowledge on the severity of symptoms.

Table 1 shows the demographic and clinical characteristics of the total sample of participants. Participants were aged between 20 and 59 years, and the majority were female (8/14, 57%). All participants fulfilled the diagnostic criteria for PTSD according to the International Classification of Diseases, Tenth Revision and had at least one comorbid psychiatric disorder. The most frequently reported comorbid disorder was panic disorder (6/14, 42%), followed by a major depressive episode (3/14, 21%) and agoraphobia (3/14, 21%), thus reflecting a population sample with complex mental health problems.

**Concepts covered in the interviews.**

**Concepts**

- Use of apps in general
- PTSD Help app utility value
- PTSD Help usefulness
- Design
- Suggestions for change
- Disadvantages or negative experiences with using the app

**Table 1:** Demographic and clinical characteristics of all participants (N=14).

Characteristics		Value	
Age (years), mean (SD; range)		39 (13.5; 20-59)	
**Gender, n (%)**			
	Female		8 (57)
	Male		6 (43)
**Diagnosis MINI^a^ (ICD-10^b^), n (%)**			
	PTSD^c^ (F43.1 in ICD-10)		14 (100)
PCL-5^d^ score, mean (SD)		46.6 (9.3)	
**Comorbidity using MINI (ICD-10), n (%)**			
	Major depressive episode (F32.2 in ICD-10)		3 (21)
	Major depressive disorder, recurrent (F33.2 in ICD-10)		1 (7)
	Agoraphobia (F40.0 in ICD-10)		3 (21)
	Social anxiety disorder (F40.1 in ICD-10)		2 (14)
	Panic disorder (F41.0 in ICD-10)		6 (42)

^a^MINI: the Mini-International Neuropsychiatric Interview.

^b^ICD-10: International Classification of Diseases, Tenth Revision.

^c^PTSD: posttraumatic stress disorder.

^d^PCL-5: The Posttraumatic Stress Disorder Checklist for the Diagnostic and Statistical Manual of Mental Disorders, Fifth Edition.

### Data Analysis

Semistructured interviews were conducted by 2 members of the research team (MEL and IMTPA) and a graduate student of psychology. Data were analyzed by first author LHGR and researcher II using thematic analysis under the supervision of an experienced qualitative researcher. Thematic analysis is a qualitative method that allows for the organization, interpretation, and reporting of data [22,23]. The analysis was conducted through 6 phases that adhered to Braun and Clarke’s thematic analysis approach [22]. The researchers LHGR and II separately coded the interviews and conducted the thematic analysis. The Lincoln and Guba [24] criteria for trustworthiness were addressed in each phase to ensure the high quality of the analysis. Through researcher triangulation, overlaps were discovered and discussed, and 3 themes were chosen. Themes were chosen according to their relationship to the overall research question.

## Results

### Overview

Table 2 summarizes the most frequently used functions in the app and the least used functions. The data indicated that all the participants except one (13/14, 92.9%) had used the app and that the mean use was 16.8 (SD 12.3) times during the study period. The total app use ranged from 0 to 39 times, indicating that some participants frequently engaged in the app, whereas one participant never used it during the study period. The most frequently used function in the app was the “Symptoms and strategies” function with a mean of 5.9 (SD 4.4). The least used function was the “Sleep Condition Indicator” with 57.1% (8/14) participants using it with a mean of 1.0 (SD 1.1), followed by the function “Positive activites” with 64.3% (9/14) participants using it with a mean of 1.1 (SD 1.1).

The following themes emerged in the thematic analysis: (1) *use of app*, (2) *being a patient,* and (3) *overall evaluation of the app*. An overview of themes and subthemes is provided in Textbox 2 and presented using verbatim quotes from the participants in the following text.

**Table 2:** Total app use and the most and least frequently used functions.

App use		Number of times that functions were used, mean (SD; range)	Endorsed, n (%)	
Total app use		16.8 (12.3; 0-39)	13 (92.9)	
**Most used functions**				
	Symptoms and strategies	5.9 (4.4; 0-15)	12 (85.7)	
	Crisis plan	5.7 (3.1; 0-13)	13 (92.9)	
	Help falling asleep	4.1 (5.6; 0-19)	10 (71.4)	
**Least used functions**				
	Body exercises	1.2 (0.9; 0-3)	11 (78.6)	
	Positive activities	1.1 (1.1; 0-3)	9 (64.3)	
	Sleep condition indicator	1.0 (1.1; 0-3)	8 (57.1)

**Themes and subthemes.**

**Themes and subthemes**

- Use of App
  - Subtheme 1: Habits
- Being a Patient
  - Subtheme 2: Negative experiences with the app
  - Subtheme 3: Being a part of a research project
- Overall Evaluation of the App

### Theme 1: Use of the App

#### Overview

The *use of the app* concerns psychological factors related to app use (such as anxiety, stress, and habits) and technical problems. The psychological factors had a dual function, as they could both motivate as well as disrupt the use of the app. For example, some participants reported using the app during panic attacks, whereas others reported that a high level of anxiety prevented them from using the app:

But when you are completely panic-struck, it's not like you think “now I'm going to use it,” it’s often like it's too late, so it is more when you are about to calm down that it is actually a help, right.

Participant ID24

Other participants described similar experiences, where even though they had used the app frequently, a high level of anxiety could prevent them from using the app. For these participants, the thought of using the app when in distress was not an option, as they simply did not remember that they had it in the exact moment:

So, do you feel that it has become a habit to use it? Understood in the sense that you remember to use it when you have a panic attack, for example?

Interviewer

No, not quite yet, but it depends a lot on how bad I’m feeling. Because if it's really bad, then it's like you don’t really think about anything, then of course I don’t think that I need to grab my phone.

Participant ID21

Some participants reported using the app to prevent stress, whereas others referred to using the app overall as a distraction tool, which was seen as a positive and useful function. A subgroup of participants experienced technical difficulties, such as missing sound on the sleep meditation or that the mobile phone automatically went to screensaver mode when they used meditation exercises, which caused some participants to stop using the app altogether.

#### Subtheme 1: Habits

The subtheme of *habits* emerged as participants described their use of the app to explain a low level of app use. One participant described how a high level of anxiety prevented the habitual use of the app, as high arousal interrupted regular use. Other participants reported that the novelty value when using the app in the beginning stimulated frequent use, but that this effect quickly vanished.

Well, of course in the beginning I used it to get to know it. And also customize it like in case of emergency, and I have also chosen my favourite functions. And I used it in the beginning, and actually got a little habit of using it a couple of times a day, but then it unfortunately went down the drain.

Participant ID14

Several participants reported either forgetting that they had the app or forgetting to use the app in relevant situations. A participant explained how he, in the beginning, tried to use the app as a distraction tool when he had a panic attack but that he quickly forgot he had the app:

You forget that you have it on your phone. Because it's not something you think about in everyday life, like “now I have the app” and then I get these feelings where I can feel all this anxiety, it's not that I go to my phone and use it, I think I just forget. That I have this tool available.

Participant ID16

### Theme 2: Being a Patient

#### Overview

The theme refers to how the patient experienced having the PTSD Help app during the waiting time until psychotherapeutic treatment started. Within this theme, the subthemes, *negative experiences with the app* and *being a part of a research project,* emerged.

For some participants, the waiting time from when the diagnostic interviews were conducted to when the psychotherapeutic treatment started felt long. One participant stated that the waiting time was too long, and he used the app as a way to not feel completely lost while waiting. Thus, the app was experienced as a supportive tool for some participants during the waiting time.

The participants also discussed their experience of participating in the initial diagnostic interview in the MHS-CRD. For example, one participant explained how there was no time in the initial diagnostic interview to explain the diagnosis, which led the patient to seek this information in the app:

...but there has not really been a time when someone has sat down with me and explained what the symptoms are, what I should expect, how the process will be, and here I think the app has been good. Because I can google everything on the internet, but here I know that it comes from a source created by doctors and psychologists. So, it's not just something that's just lying around on the internet.

Participant ID39

On the other hand, some participants reported being overwhelmed by the status of being a patient. Having to use the app added to the stress. A participant explained how facing PTSD-related problems would make things worse, and that she did not feel able to engage in the app unsupported:

So, from the very first interview, it took almost a month before I had the next two appointments. And during that time, I shut down everything, and didn’t want to relate to anything, or think about anything, and I pushed everything away, and when there were four-five days until I had to start, everything got worse up to the maximum again. I only slept two-three hours a night and woke up with anxiety and palpitation and...yes. So, the bottom line is that when I'm forced to deal with it, it gets worse.

Participant ID29

The participants who addressed the theme of being a patient all highlighted the waiting time as problematic and that information (either from the app or from a professional) about their diagnosis and the overall treatment process were helpful.

#### Subtheme 2: Negative Experiences With the App

Although there was an overall positive attitude toward the app in that participants reported becoming more aware of symptoms and sensations that increased their understanding of the disorder, negative experiences from using the app were also reported. Although participants generally saw becoming more aware of their symptoms as clinically beneficial, some experienced it as a worsening of symptoms and exposing vulnerability in a situation of minimal support. One example of a participant reporting using the app regularly reported feeling worse from the increased self-awareness that followed using the intervention tools in the app:

So, you may have become more aware of yourself if you can put it that way?

Interviewer

Yes exactly, and what to look for to why you have these symptoms, and what these symptoms mean and that it is not, you know, your own fault, if you can say so.

Participant

Have you noticed if any of your symptoms have changed since you got the app?

Interviewer

I do not think changed, I just think they have become...all in all, worse.

Participant ID39

The participants who reported negative experiences all agreed that focusing on their PTSD symptoms without support from a professional caused increased distress.

#### Subtheme 3: Being a Part of a Research Project

Some participants reported how being a part of a research project was stressful:

But what I would also like to say is that I have been to a psychologist before, and now I go to the psychiatric clinic in Hilleroed to find out what treatment I should have, and then at the same time, I should also use the app, and at the same time you get calls, and I get asked a lot of things about the app, so I just think...I'm well aware that you have some of these things, but it might be a little...too much, then I have to use the app, then I go to a psychologist, so it might also take up a little too much time in everyday life, I think...That you are constantly made aware that you have this problem.

Participant ID16

Another participant felt almost the opposite, as he explained how contact with the research team helped him relax as he knew he could reach out to the research team if he needed help.

### Theme 3: Overall Evaluation of the App

This theme covers how the participants experienced using the app, the design of the app, and its specific functionalities. Within this theme, participants provided several suggestions for changes to the app.

Overall, most participants reported using the app regularly and were generally positive toward it. The design of the app was reported to be accessible and easy to use, although some participants reported that a few of the functionalities, such as the location of the questionnaires, were not placed intuitively and were therefore confusing. The participants had several suggestions for improvements, such as implementing notifications as reminders, providing psychoeducation for relatives, and personalizing the app.

...you’re able to personalise the app, where you can put some pictures in it that make you happy, or something like that, right. Because I have learned that on my computer, when it‘s in sleep mode, it shows some pictures, and for me it has actually been really relaxing to be able to sit there and watch these pictures and think “okay.” So, something like that, I don’t know...you just relax when seeing something of your own that you have chosen in advance, and maybe also some music that you might like or something like that.

Participant ID24

In particular, the suggestion to implement notifications in the app as a reminder for using the exercises, self-assessment, and other features was mentioned several times. There was a broad consensus across participants that the key advantages of the app were that it was a reliable source of information on PTSD and that it worked well as a distraction tool. A small number of participants reported being surprised at how effective some of the emotion regulation tools were (eg, the soothing images).

## Discussion

### Principal Findings

This qualitative study explored patients’ experiences with using the PTSD Help app as a stand-alone treatment before commencing psychotherapeutic treatment. All the interviewed participants except one (13/14, 92.9%) had used at least some functions in the app with the range of use among the participants from 0 to 39 times during the study period. Thus, nearly all participants had experience from engaging with the app to report. The data also indicated a broad range of use among participants.

A total of 3 themes were identified in the analysis*— the use of the app*, *being a patient,* and *the overall evaluation of the app*. Overall, the participants felt comfortable using the app and experienced several functions in the app as helpful. However, the analysis found that a subgroup of participants reported negative experiences with the app. Finally, the wish for a more personalized app emerged.

Our findings showed that some participants reported that increased emotional arousal (eg, anxiety) could lead to both an increase and a decrease in app use depending on the situation. Thus, an optimal level of distress seemed to promote the use of the app and increase individuals’ ability to use emotional regulation strategies when needed. One could speculate that to remember to use the app efficiently, at least some distress is necessary. This issue is important to note, as it raises the question of the existence of an optimal load of symptoms when using an app as a stand-alone treatment. Previous studies on the effectiveness of mobile apps have focused on symptom improvement rather than patterns of use [6]. Our study suggests that it is possible that patients who are less impaired by their symptoms will benefit the most and be the most engaged users of the app, whereas patients with more severe symptoms will, to a higher degree, refrain from using the app.

What is new is our finding that a subgroup of participants reported negative experiences with the app. Although this applies only to a limited number of participants, it must be taken seriously, as increased symptom severity in PTSD has been associated with increased suicidal risk [3,4]. This finding emphasizes the potential general challenge of app interventions as a stand-alone treatment. In contrast to psychotherapy, where the clinician’s continuous evaluation of the patient, including risks, benefits, and goals, guides which intervention is used, mHealth apps offer the same functions to all patients, potentially leaving vulnerable patients unassisted in choosing and applying interventions [25]. Although the PTSD Help app offers a crisis management plan and strategies for coping with suicide risk, the use of these functions is dependent on the patient, who may have limited insight and, as such, risk not using the functions when needed or not being able to use the functions unassisted. Consequently, it could be argued that to be able to support this vulnerable subgroup of patients, a blended care intervention, including clinician support, is crucial.

Habits were raised as a subtheme in the way the participants used the app. As using the app is a prerequisite to obtain an effect, it is relevant to understand patterns of use and factors involved in the participants’ use of the app. One perspective on patterns of use comes from research on habits and habit formation [26]. Developing habits is a dynamic process in which behavioral control is initially goal dependent but shifts to context dependence as the behavior is repeated [27]. In our study, the participants were responsible for defining their goal on their own and received limited contextual instructions in using the app, and the instructions were somewhat vague (eg, ‘when you are feeling distressed’). The analysis found that participants reported how forgetfulness and the use of the app not becoming habitual caused unintentional nonadherence. Accordingly, a recent systematic review focusing on medication adherence across chronic medical conditions found that habit strength was strongly correlated with medication adherence [28]. Focusing on psychotherapy, a study on a guided self-help intervention using cognitive bias modification training found a significant treatment effect in patients with depression who reported having created a habitual use of the practiced self-help response [29]. These findings suggest that supporting habit formation through clinician-supported interventions on goal definition and contextual instructions appears to be important when implementing an mHealth app and could have had an impact on the frequency of use of the PTSD Help app.

In relation to the previous suggestion to use a blended care approach to support patients with potential suicidal thoughts in using the app, a blended care approach could also strengthen habit formation and thus potentially be of more help. This would also apply to vulnerable patients with an increased risk of suicide. If using the app successfully becomes a habitual behavior, it will automatically be activated in the relevant context and, therefore, not be dependent on available mental resources, which may be limited in situations with high emotional arousal.

This study also revealed how the waiting time until treatment starts could feel long and stressful for the patient and result in the patient not engaging in the app, and clinician support could alleviate this. Previous studies have shown that including clinician support in internet-based treatments increases the effect size and patient adherence [30,31]. A study on a clinician-supported version of the PTSD Coach app found promising results in terms of acceptability and feasibility among patients and clinicians [32]. The procedure in the present study did not include clinician support, but it included a technical support call after 3 days, which some participants perceived as a clinical support call, which in turn might have encouraged participants to be more engaged in using the app after this call. Our findings suggest that providing participants with more contextual instructions and at least some clinician support on a regular basis may be important to make the use of the app habitual, which could prove pivotal in optimizing the effect of an app.

The participants’ evaluation of the app was closely related to suggestions for change, in which a wish to be able to personalize the app was prominent. This request is consistent with previous qualitative studies on the internet and mobile-based interventions [33,34]. Although the app offers personalization of the content to some extent, this finding echoes the need for more personalized material and interventions. With the PTSD Help app, this could mean changing the focus from the individual choosing his or her own preferred functions in the app to the app recommending relevant interventions based on a data collection of the individual’s specific distribution of symptoms. As the app already contains self-monitoring tools, in line with recommendations [9], it seems plausible to use this collected data in an automated tailoring algorithm. A variety of functions should be offered in the app to ensure that it holds content of relevance to every participant with a variety of individual needs and preferences.

Regarding age, all the participants were aged ≤60 years, making it difficult to generalize the results past that age. However, the majority of patients treated in MHS in Denmark are aged ≤60 years, as demonstrated in demographic data from a large sample of 2473 patients without a diagnosis of psychosis in MHS-CRD, where the average age was 33.0 years [35]. Thus, our results cannot be generalized to older adults but can mirror the average age seen in MHS in Denmark.

### Limitations

Some limitations influence the strength of the conclusions drawn from the results of this study. The sample was based on a convenience sampling principle, because of a strict time limit for recruitment. The most optimal sampling strategy would have been a selected sample, which would have allowed the information from this study to be applied beyond its settings [36]. Furthermore, no participant in the study was older than 59 years, making it difficult to generalize our findings to older adults.

There is a possibility that there was a positive sampling bias, as those participants who agreed to participate generally had a positive attitude toward the app. Two participants were asked to participate but declined because of a reported negative attitude toward the app. One could speculate that this positive attitude is not necessarily the general attitude of all participants using the app. This could also explain how the data on the total app use showed that one participant did not engage in the app at all during the study period, but this was not revealed in the interviews.

The study covered participants’ experiences of using the app and acknowledged that there are multiple factors, such as technological, psychological, and social factors, which affect the experience. These factors may not be fully covered by the semistructured interview in this study, as they are not always apparent to the participant or the participant may not wish to reveal them to a member of the research team.

### Future Implications

In light of the current COVID-19 pandemic and the urgent need for developing internet-based treatment services that ensure treatment commitment and adherence, it is important to cover the patient’s experience of using mHealth apps, as this will provide the research field with essential information about usability, acceptability, and possible negative effects. This qualitative study makes an important contribution to the field of mHealth research, showing that providing an mHealth app to patients with PTSD without clinician support should be done with caution. Our findings stress that personalization and active interactions could be beneficial to integrate in future mHealth apps to ensure continuous engagement and positive experiences with the treatment. On the basis of these findings, we provide two suggestions when implementing an mHealth app in an MHS with a PTSD population: (1) using a blended care treatment with clinician support to provide assistance and regular psychiatric assessment as well as to stimulate habitual use and (2) using an approach where mental health care professionals *prescribe* an mHealth app for relevant patients. Using one of these suggestions when implementing an mHealth app for patients with PTSD in MHS will enable clinicians to identify patients who are at risk of experiencing worsening symptoms from using the app unassisted. Future studies could investigate the association between use and outcome to determine if a dose-response mechanism exists and which functions patients seem to engage the most with signaling, which might be more important than others.

### Conclusions

This qualitative study examined how patients with PTSD experienced the mHealth app PTSD Help as a stand-alone treatment before commencing psychotherapeutic treatment. The overall findings were that the majority of participants expressed that they had a positive experience using the app, but that a subgroup of participants reported negative experiences using the app. The lack of habitual use of the app was also raised to explain a low level of use and not using the app in relevant situations.

There are undoubtedly many advantages in implementing mHealth apps in MHS. Our findings demonstrate the need for research on which patients can benefit and which patients are at risk of experiencing worsening of symptoms from having an mHealth app as a stand-alone treatment. We hope that this study will serve as a springboard for future mHealth studies to form hypotheses about which patients in particular can benefit from using an mHealth app (either as a stand-alone treatment or as a supplement to treatment) and explore this in a mixed methods approach to provide new fine-grained insights into the research field. As a consequence of the participants’ wish for a more personalized app, we also encourage further investigation of the possibility of developing such an app using a tailoring algorithm to recommend interventions to the user.

## Abbreviations

DSM-5: The Diagnostic and Statistical Manual of Mental Disorders, Fifth Edition
mHealth: mobile health
MHS: mental health services
MHS-CRD: Mental Health Services of the Capital Region of Denmark
PTSD: posttraumatic stress disorder
